# Anticipated stigma and blameless guilt: Mothers' evaluation of life with the sex-linked disorder, hypohidrotic ectodermal dysplasia (XHED)

**DOI:** 10.1016/j.socscimed.2016.04.027

**Published:** 2016-06

**Authors:** Angus Clarke

**Affiliations:** Medical Genetics, Cancer & Genetics, School of Medicine, Cardiff University, Wales UK

**Keywords:** United Kingdom, Stigmatisation, Discrimination, Guilt, Blame, X-linked hypohidrotic ectodermal dysplasia, Grandmother, Reproductive decision

## Abstract

Practical experience of a genetic disorder may influence how parents approach reproduction, if they know their child may be affected by an inherited condition. One important aspect of this practical experience is the stigmatisation which family members may experience or witness. We outline the concept of stigma and how it affects those in families with a condition that impacts upon physical appearance. We then consider the accounts given by females in families affected by the rare sex-linked disorder, X-linked hypohidrotic ectodermal dysplasia (XHED), which principally affects males but can be passed through female carriers to affect their sons. The stigmatisation of affected males is as important in the accounts given by their womenfolk as the physical effects of the condition; this impacts on their talk about transmission of the disorder to the next generation.

Perspectives may also change over time. The mothers of affected sons differ from their daughters, who do not yet have children, and from their mothers, who may express more strongly their sense of guilt at having transmitted the condition, despite there being no question of moral culpability. We conclude with suggestions about other contexts where the possibility of stigma may influence reproductive decisions.

## Highlights

• Living with XHED presents physical and social challenges, including stigmatisation.
• Female carriers observe the stigmatisation that impacts their affected male relatives.
• Mothers expect that an affected child may be subject to stigmatisation.
• Such anticipated stigma impacts the way female carriers talk about reproduction.
• The perspectives of sisters, daughters, mothers and grandmothers on XHED may differ.

## 1 Introduction

Reproduction is an important aspect of personal identity, especially in families impacted by genetic disease, where the risk of transmitting an inherited disorder to one's children is not merely a biological but also a social fact, with important consequences for relationships and family life. These consequences vary not only with the culture but also with the mode of (biological) inheritance and the age and manner in which the condition becomes manifest.

We have previously shown that males affected by a rare sex-linked disorder, X-linked hypohidrotic ectodermal dysplasia (XHED), experience stigmatisation and that this impacts on how they view their lives. This in turn has implications for their attitudes towards transmitting the condition to future generations (Clarke, 2013). Affected males often encounter serious health problems in early childhood including overheating (from inability to sweat), recurrent chest infections, allergies and failure to thrive. While some succumb, the majority survive and learn to manage these physical challenges (Clarke et al., 1987), with the survivors often reporting stigmatisation for their physical (especially facial) features.

In this paper, we outline the concept of stigma as it affects those with visible physical difference and consider the types of stigmatisation that can operate in XHED families. We then report the accounts given by female carriers of XHED, who usually show few signs of the condition but may have severely affected sons. Their accounts of how XHED impacts on the affected males suggest that it may also influence how they make reproductive decisions. We conclude with a consideration of other disease contexts in which the anticipation of stigma may influence family reproductive decisions.

## 2 Stigma and inherited disorders

Goffman's concept of stigma as “an attribute that is deeply discrediting … reduc[ing] the bearer from a whole and usual person to a tainted, discounted one” (Goffman, 1968) is key to our understanding of stigma. He distinguished the enacted stigma associated with overt physical difference from the potentially discrediting stigma associated with information that may become known about a person or some physical attribute that can usually be concealed. Courtesy stigma was described by Goffman as the stigmatisation of those who associate with the stigmatised. This often arises when family members accompany an affected individual in public. The concept of stigma has developed since then to incorporate a wider range of experiences. Those with epilepsy anticipate the stigmatisation that may follow a seizure witnessed by others: this has been termed ‘felt stigma’ (Scambler and Hopkins, 1986). A similar anticipation of stigmatisation arises in relation to other triggers, such as mental illness and HIV infection. Internalisation of this feeling – when the individual accepts the stigmatisation as justified – contributes the internalised aspect to the triad of internalised, interpersonal, and institutional stigma that links stigma with discrimination (Jacoby and Austin, 2007). The shading of stigma into discrimination (Pescosolido et al., 2008) applies especially in the context of mental illness, HIV infection and race, when the 'Other' may be seen as a threat. Felt stigma arises in many other contexts, including obesity, where the distinction between felt and enacted stigma often blurs, manifesting as hypersensitivity and an over-interpretation of others' speech and behaviours (Barlösius and Philips, 2015). Stigmatisation of this type may often be reinforced by the behaviour of health professionals (Hamlington et al., 2015).

Conceptual analyses of stigma have especially addressed mental illness and HIV infection, and some aspects may not apply to the original context of overt physical difference. Thus, Link and Phelan (2001) define stigma as the co-occurrence of five elements: labelling, stereotyping, separation, loss of status and discrimination. This entails a difference in power between the stigmatised and those who stigmatise them, a clear feature of the experience of those with HIV and mental illness.

Those with physical blemish may initially be thought to fit less readily into this category of being a threat, although the power asymmetry between the ‘stigmatised’ and ‘normal’ may be very real. Rather than fitting a stereotype, the ‘blemished’ fall short of the generally accepted standard of attractiveness. This restricts opportunities in many categories of employment as well as denying full participation in social life.:the power involved in this discrimination is not monolithic but diffuse, and operates locally.

What difference is made by the genetic origin of a stigmatising condition? If a condition is biologically disadvantageous and genetic in origin, it could be seen as a threat to the community. Stigmatisation of those with physical blemishes, rather than simple physical difference (e.g. skin colour), may then have a Darwinian origin, making it less likely for the individual to be socially accepted (Kurzban and Leary, 2001). However, as with much ‘evolutionary psychology’, such unfalsifiable speculation may be best ignored.

There is a complex relationship between an identified genetic cause for a disorder and feelings of stigma (Sankar et al., 2006), so that a genetic origin for some conditions may bring relief from stigma because they are not contagious while, in the case of certain cancers, a genetic cause may be feared if it suggests a worse prognosis. Similarly complex responses arise to the suggestion of a genetic cause for epilepsy (Shostak et al., 2011). Interestingly, the mode of inheritance of a disorder has been found to have a major impact on feelings of guilt and blame, with female carriers of sex-linked disorders feeling more guilt and being subject to more blame than mutation carriers in autosomal disorders (James et al., 2006). Furthermore, those female carriers of severe X-linked disorders who have close personal experience of life with an affected brother engage more concretely with the issues, feel more guilt and express a greater sense of the responsibility entailed in making reproductive decisions than those who have not had such experience (Kay and Kingston, 2002). These findings are reflected in other accounts of life in families with the sex-linked disorder Duchenne muscular dystrophy (DMD), such as the study of Parsons and Atkinson (1993), where the sisters of affected males often expressed the view that they could only ever have daughters (meaning that they would terminate a pregnancy carrying a male foetus at risk of DMD. Definitive prenatal diagnosis was not yet available when these families had been interviewed).

The stigmatisation of carriers has also been recognised in the context of other disorders, often in association with the view that the making of decisions about reproduction is a weighty matter and that disease carriers should do this in a responsible manner. In the context of screening for autosomal recessive disorders, this has been demonstrated for sickle cell disease (Kenen and Schmidt, 1978) and Tay-Sachs disease (Zeesman et al., 1984) among others. Similarly, those making antenatal screening programmes available – both the policy makers and at least some health professionals – have an expectation that pregnant women will (and, by implication, ‘should’) participate in screening designed to identify pregnancies in which the foetus has Down syndrome and/or a serious malformation. The pressure exerted in such screening programmes, arising from the very existence of the screening programme, can amount almost to coercion, whatever the wishes and intentions of the individual professionals involved (Clarke, 1991, Clarke, 1997, Rothman, 1988, Rapp, 2000).

More recently, the meaning of responsibility in relation to genetics has broadened very considerably as the scope of applicability of genetics has broadened from its earlier focus in the sphere of reproduction to include the whole breadth of medicine and healthcare more generally (‘mainstreaming genomics’). The original idea of geneticisation as a critical concept, questioning the value of a focus on the genetic aspects of disease as a distraction from the more important and more tractable non-genetic factors (Lippman, 1991) is no longer so readily applicable because clarification of the relevant genetic factors may be an essential preliminary to defining and woking with the other, non-gemetic causal factors. In fact, the genetic factors may be more tractable to analysis than the chaos of nutrition and behaviour, and disease is seen often to arise from their complex interaction. While Kenen was correct to focus on the difficulties of working with probabilistic factors in causation and on the difficulty of maintaining the privacy of personal genetic and health information, as those issues remain highly relevant today (Kenen, 1994), it would be untenable to turn away from the powerful insights into disease mechanisms afforded by genetic approaches.

The broader sense of genetic responsibility, and the responsible use of the new reproductive technologies within it, has been examined by a range of authors, including – prominently – Novas and Rose (2000). The associated discourse of genetic responsibility employed in the clinical setting has also been examined and dissected (Arribas-Ayllon et al., 2011).

## 3 Stigmatisation in XHED

Families experience both the practical physical effects of ‘their’ condition and its social consequences, especially stigmatisation, and the mode of inheritance interacts with both of these. Such interactions are complex, as envisaged by Goffman when he wrote, “And where stigmas are very visible or intrusive, or are transmissible along family lines, then the resulting instabilities in interaction can have a very pervasive effect upon those accorded the stigmatised role” (1968 p.164). This paper can be regarded as an extended footnote to this remark. The condition XHED meets both criteria for 'instabilities in interaction', as the condition is inherited and the physical (especially facial) features often lead to stigmatisation.

This paper examines the accounts of women in XHED families, who have usually observed the physical and social impact of XHED on their affected menfolk over years. The women may be subject to some stigmatisation, such as courtesy stigma from association with their affected male relatives, and they witness their fathers', brothers' or sons' enacted stigmatisation on account of their discrediting physical appearance. Affected males may experience felt stigma from anxiety in case of discrediting episodes of heat exhaustion (with transient inability to function in certain social roles, especially employment), comparable to the felt stigma of epilepsy. Female carriers will occasionally be subject to direct stigmatisation if they happen to manifest the condition to a marked degree from an unfavourable pattern of X chromosome inactivation.

Another type of stigmatisation that impacts upon affected males, their parents and their female relatives is a *trans-generational* form of *anticipated*, or felt, stigmatisation, when the risk of having an affected child counts against the person's standing as a potential partner or parent. Such stigmatisation on the grounds of 'inferior reproductive worth' may be enacted for the males but discreditable for the carrier females. Judgements of reproductive worth are familiar from 19th and 20th century eugenics but have not often been studied in relation to particular inherited disorders. A case report describes discrimination against a woman on the grounds of disfigurement from a genetic disorder, neurofibromatosis type 1 (Rozario, 2007). Goffman perhaps referred to such judgements when he stated, “the devaluation of those with bodily disfigurements can perhaps be interpreted as contributing to a needed narrowing of courtship decisions,” (Goffman, 1968, p. 165) although his moral stance is unclear.

Reproductive stigmatisation impacts differentially on the affected male, his parents and on female relatives. The affected male will not have affected sons but his daughters will all be carriers; it is the female carriers whose sons have a 50% chance of inheriting the condition as the condition is sex-linked. His parents may be blamed by others for having an affected child, especially a second or third affected child. The man himself may be regarded as unfit to have children in case they are affected, although that would reflect a misunderstanding of the inheritance pattern. Those female relatives who are carriers will have a 25% chance in each pregnancy of the child being an affected male, and may therefore experience this trans-generational or reproductive stigmatisation.

The goal of this paper is to open up for discussion the potential instabilities discussed by Goffman, demonstrating these complexities in the context of HED. Such instabilities have been documented in the work of Boardman, 2014a, Boardman, 2014b in relation to the neuromuscular disorder, spinal muscular atrophy, although that shows a different pattern of inheritance.

To what extent can ‘looking different’ itself count as a disability, even if there are no associated medical problems? If someone were judged as unfit to have children because they were thought to be ‘ugly', or because their children were likely to be ‘ugly', how would this relate to the discrimination against those judged as unfit to have children because they may have a physical or cognitive impairment? One important difference is that systematic discrimination against the latter is often mediated by antenatal screening for foetal abnormality and that is not relevant in the context of ‘ugliness', or of XHED. Again, popular stereotypes exist for some of the conditions 'sought' in antenatal screening programmes but not for ‘ugliness’ or XHED. However, there may be strong parallels between the feelings aroused by judgements made within the family and the feelings manifest in the expressivist objection to antenatal screening programmes as an assertion of their human worth (Parens and Asch, 2000).

## 4 Methods

Families with XHED were recruited both through previous contact (an earlier clinical study) and through the UK family support organisation, the Ectodermal Dysplasia Society (EDS). More details of the research context, recruitment process and analytic methods are given in the previous report on the affected males' accounts of stigmatisation (Clarke, 2013). The fact that many participants already knew the interviewer in a different role (biomedical researcher) can be seen as a strength or a weakness: on balance, we see this as a strength as discussion could move easily to salient points as informants could assume background knowledge about the condition and their family situation instead of having to explain the context from scratch. Members of twenty UK families were interviewed between April 2001 and January 2008, most before July 2004, with consent according to the terms of the project approval by the appropriate NHS Multicentre Research Ethics Committee. Fifteen interviews included mothers, with or without additional family members present; they were informal, in the family home, and the family functioned 'around' the interview, with some shifts in who was participating. For fourteen of these interviews we have transcripts of audio recordings and for one a written record from field notes (when the recording failed). We focus on the contributions of the female carriers but include contributions from others present.

There were approximately 80 invitations to participate in the research distributed in an issue of the newsletter distributed to families by the (UK) Ectodermal Dysplasia Society. There were approximately 40 letters sent to families already known to the author, many of whom will also have received the invitation through the EDS newsletter. Only three of the 20 families who took part in interviews were not already known to the author; the mothers of affected males took part in two of those three ‘previously unknown family’ interviews and so they constituted 2/15 interviews considered in this paper. We cannot present an accurate participation rate for either mode of invitation but the response rate among families already known to the author was approximately 40%.

Contact with the families already known to the researcher was most often through the mother of an affected male, who had usually been a child in the early 1980's. The primary contact in these families was therefore usually the mother of a young man in his 20's, or older, at the time of these interviews. Practical arrangements were therefore focused on access to the young man and his mother. When sisters were included in the discussion, this occurred by chance or when the family had decided to arrange this, as we had no direct contact with these young women.

The goal of reporting these interviews in publications was explicit throughout and was included in the processes of research ethics approval and participant consent. Some minor details have been modified in a few transcripts, concerning family structure, geographical location or the nature of other diseases present in the family, to protect confidentiality; care has been taken that this does not affect the social context or meaning of the interviews.

The transcripts were read repeatedly and the topics addressed were mapped out. Key points in each interview were analysed in more detail, as in the excerpts given below, where the detail of what was (not) said has been used to infer underlying emotions, assumptions and motivations. The research perspective is that of a materialist understanding of biology, a constructivist understanding of social life, and a rhetorical discourse analytic approach to the understanding of interviews as accounts (Arribas-Ayllon et al., 2011). This acknowledges the situated nature of such interviews, in which the interviewer will be concerned to present himself as a competent, caring professional interested in the experiences of affected individuals and their families, while the interviewees will (usually) wish to demonstrate effective coping with XHED while managing their family lives in a manner that is both emotionally satisfactory and morally responsible.

The topic that demonstrated most difference between interviews was that relating to reproduction. The interviewer was sometimes reluctant to raise this topic directly because of the wish to be tactful and show respect for the research participants. Direct questions about reproduction were employed selectively, sometimes being deliberately avoided as illustrated in this interview:

“The key moment … was when [Mother] expressed her guilt at having passed on the burden of being a carrier to at least [Daughter 1] even if not [Daughter 2]. She knows she has done nothing blameworthy but her natural desire to protect her children makes her feel responsible …. She feels worse about this than about transmitting the condition itself to [Affected Son]. Both her daughters say they do not want to have children – they have seen how tough it has been for their parents (Mother's eyes moistened). I could not bring myself to ask how she would advise a young couple at risk of having an affected child to act: would she recommend a termination of pregnancy for an affected son? or even for a carrier daughter?”

(Field note to Interview 8).

## 5 Findings

The findings will be presented in three sections. We first report the concerns many mothers feel about the stigma experienced by their affected sons. Next we give a brief overview of the statements made by the mothers of affected males concerning the transmission of XHED to their children in relation to prenatal diagnosis and the selective termination of affected pregnancies. Finally, we present some more detailed, discursive accounts of the views and experiences of these women under several headings. (Note the use of = to indicate overlapping turns in the transcription).

### 5.1 Concerns about stigmatisation

When asked about the impact of XHED on the lives of the affected males in their families, mothers give as much emphasis to its effects on social life as on coping with heat intolerance and other physical effects. Accordingly, we report some of the concerns about stigmatisation from three interviews.

This mother recalls her adult son as a young child:

**Table:** 

Mother:	… but when he was small he used to come in crying at the kids, I mean his hair, his scalp used to shine through his hair, he didn't have normal hair ….

(Multiple turns omitted)

**Table:** 

	Yes, I used to say to him [Name of Son] “Look, sticks and stones, never hit them!” and “You are as good as any of them, you know” and I used to say, “Just try not to let them bother you; you have got your cousin” I mean his cousin used to stick up for him …. but he would still come in sometimes crying.

Interview 13A (Mother of Affected Male).

Another mother reports having worried about her son being bullied or socially excluded during his school years:

**Table:** 

Mother:	Um, that was always my biggest worry really. Bullying – when he started school that was a huge worry for me. That was one of the things I …. . I worried really about.

(Several turns omitted)

**Table:** 

Mother:	Would he have any friends? Would he ever get a girlfriend? That kind of thing, you know. Would he have a normal life? That was my biggest worry. ….

(Interview 9. Mother with her affected son present, aged 15 years)

Another mother reported distress in her 16 year old son when passers-by commented on his appearance:

**Table:** 

Mother:	He came home … and was a bit quiet in the evening and then we had a chat about it … and he actually did have a little tear about it, ‘cause he said, “Do I look awful?”, this was only a couple of months ago. “Well”, I said, “no you don't!”. We always start with, “You've got two arms, two legs, a good brain. I know he hurt you but if you let it get to you”, that's what I always say, “If you let it get to you, it will just affect all your life all the time. So you've just got to pick yourself up and think, “That was unfortunate that chap said that but he didn't really mean any harm by it – he was just being insensitive”.

Interview 1. Mother of 16 year-old affected male (no longer present).

Such accounts show how female carriers of XHED learn about the social as well as the physical effects of the condition.

### 5.2 Overview of the statements of mothers and grandmothers about reproduction and the use of prenatal diagnosis and the selective termination of pregnancies

Of eight mothers who expressed an opinion, four were simply opposed to prenatal diagnosis (PND) and the selective termination of affected pregnancies (SToP), often having undertaken pregnancies where they knew a son would be at 50% risk of XHED. One mother was opposed to PND/SToP but with some ambivalence (her view was complex and nuanced). Two mothers were ambivalent or undecided: one indicated that she would not use such technology but could understand – and would not blame – anyone who did; the other reported that she had terminated a pregnancy at risk of being affected because she was subject to pressure from health professionals.

One woman, the mother of two affected males and grandmother of another, expressed guilt and regret at having transmitted the condition to her children and grandchildren. Another grandmother, thought to be a carrier, was reported by her daughter to express similar feelings.

### 5.3 Reproductive guilt and prenatal diagnosis

Several mothers of affected males reported feeling guilt, or at least regret, that they had transmitted the XHED to their affected son. An example is this simple expression of feelings:

**Table:** 

Mother:	I feel guilty that I've landed him with something that's making him ill …

Multiple turns omitted.

**Table:** 

Mother:	It's hard to define whether I'm feeling upset when he's poorly because he's just poorly or I'm upset because I feel I made him =
Int.:	=You feel responsible
Mother:	poorly

(Interview 18 – Carrier Mother and Affected Son, age 21 years)

This woman focuses on the physical challenges of XHED and expresses guilt that she has transmitted this to her son. The mother of another affected male expresses guilt but presents this as a feeling from the past:

**Table:** 

Mother:	No. I mean I know I went through a guilt stage. That I felt guilty that [Name of Affected Son] had got ED. But I think that was more when he developed Crohn's disease as well because I thought that was so unfair. I thought he'd got one thing=
Int:	=Yeah.
Mother:	why does he need (.)
Int:	Yeah.
Mother:	something else? But life doesn't always give you what you think you …. deserve

(Several turns omitted)

**Table:** 

Int:	Yeah. (.) You mentioned the word 'guilt'
Mother:	(hmm)
Int:	which I know, you know, people do sometimes feel=
Mother:	=guilty
Int:	if their child has a problem that you can trace back in the family
Mother:	Yeah.
Int:	they do sometimes feel that.
Mother:	Yeah.
Int:	and you can (.) there are quite a few (.) there are lots of different ways of look ing at it, aren't there? But is that (..) you say you had “a phase” of that
Mother:	((hmm))
Int:	I mean is that you still feel (.) or is that something that you can say, you know, “okay, that happened” but, you know, looking rationally you can see there's no sense in which you are to blame. Can you=
Mother:	=I can now
Int:	look at that and say I'm not, you know, morally (.) whatever?
Mother:	Yeah. I can now 'cause I say, again, because we didn't know (.) we knew there was something in the family but it was like (.) it had never been a (.) really it had never been an issue in the family.

(Interview 6. Mother of 27 year old affected son, who was present)

It has required the passage of time but the mother asserts that she can now distance herself from the guilt she used to feel at having had an affected son. In the presence of her son, however, it might have been difficult to express persisting regret at having given birth to him.

Later in the same interview it became clear that there was still strong emotion attached to the diagnosis, relating to the question whether the condition is sufficiently serious to warrant SToP. This discussion occurs in the presence of the (adult) affected son, and it is perhaps his presence that leads to embarrassment. The mother explains that she would never terminate a pregnancy affected by XHED, although there was awkwardness, manifest in dysfluency, when she accounted for this by explaining that XHED is milder than some other conditions such as cystic fibrosis (CF). This contrast relies for its success on the implication that CF would be generally accepted as sufficiently severe to warrant a termination of pregnancy. The emotional stakes are raised as the interview continues:

**Table:** 

Mother:	I mean, if I had known about ED
Int:	Yeah.
Mother:	before I'd had [Affected Son] like they can do tests now and somebody
said	“well, your child has got ED. What do you want to do? Do you want to carry on with this pregnancy …”
Int.:	Yeah
Mother:	“ … or do you want to terminate?” then I would always carry on because [Affected Son] has given us (.) so much – I'm sorry [Affected Son]! ((laughs)) – no, but he has and I don't think it's (.) it's not like a cystic fibrosis or do you know what I mean?
Int:	(hmm)
Mother:	Do you know what I'm trying to say to you?
Int:	Yes, yes.
Mother:	It's not like that sort of condition.
Int:	Yeah.
Mother:	Oh, I don't think I'm expressing myself [very well]=
Int:	=[No, I think] I think you are.
Mother:	Do you know what I mean? So that (.) therefore, (.) oh dear (.) you know (.) … (it does depend) … on the severity …

This mother's dysfluency could relate to discomfort either with her assumption that CF is so much more severe than ED or with the very act of discussing terminations of pregnancy for XHED in front of her son, despite her clear rejection of this as something she might choose. The strength and persistence of the dysfluency would be unlikely if the former explanation were correct, so it is more likely that discussion about a termination of pregnancy for XHED in the company of her son triggered the disfluency.

In the next passage, another mother explains that she would not terminate a pregnancy on the grounds of XHED but can accept that some women might. She acknowledges and, in doing so, simultaneously demonstrates how complex these decisions can be:

**Table:** 

Int:	thinking about, in some families, thinking about terminations and so on …
Mother:	Yes
Int:	because of it …. then that's something that's much harder. Do you think … have you got, sort of, thoughts and feelings about all that?
Mother:	Well (.) whatever [Daughter] decides will be her decision. I wouldn't want to sway her either way. There's no way I would not have had [Name of Affected Son] personally because I would hate to have missed out on his life. And it's – I don't want it to sound like I'm skipping through corn fields – but our life has been very rich having [Affected Son], very difficult at times, but if someone felt that they had to terminate because of that then … I would never hold that against [Name of Daughter] if she decided that. I think I'd just have to distance myself from it=
Int:	=Yeah.
Mother:	… and think it's that person's decision=
Int:	=Yeah
Mother:	and what they do is up to them. Knowing [Affected Son], I would never have a termination. Had I not known anything about it, or anything, and I was a lot younger I might have had a different view. But if someone had said to me, if someone had … said to me “this is my son and your son is going to be like this” and he was a lovely strapping lad who had got safely to that age, I would have been OK. I think it's fear of the unknown really. But, I mean, I've known people who've had abortions for no reasons at all, other than they don't want the child ….
Int:	I'm sorry to bring it up in a sense.
Mother:	Yeah … No, no – it's just something that is, well, other people think about a lot more than I do. I don't know how [Affected Son] would feel if he thought [Daughter] might have a termination because she might have a child like him.

(Interview 1. Mother of teenage affected son, 16 years, no longer present in room.)

This passage demonstrates how the interpersonal implications of an individual's decisions are recognised as having potentially profound repercussions (“instabilities of interaction”) within a family, with the capacity for a mother's words or sister's actions to cause hurt to an affected male. Furthermore, it is clear that this potential is taken into account by the mother as she discusses the condition. She makes a clear link between knowing her son and her judgement that she would not want to terminate an affected pregnancy. This amounts to a ‘vote of confidence’ in the value of her son's life. The fact that this judgement is retrospective is important, because her daughter (if she were making a decision about a pregnancy) would inevitably be faced with ignorance about how the life of her affected son would work out; she might feel a 'fear of the unknown'.

In another interview, a carrier mother expresses similar conclusions: she would accept the risks of having (another) affected child and hopes her daughter would do the same:

**Table:** 

Int:	If any of you are talking to a couple who are, who might have a child with this, what sort of things would you be saying?
Mother:	I'd say get in there and have a child and you know worry about it if it happens. It might not. That would be my best … You know we've got to go through this with [Daughter] yet. And I would hope that she would not see it as a problem. Because it hasn't been.
Int:	Mm. Well it's been a problem in some ways.
Mother:	Well yes but it's not such an insurmountable, incurable problem that (.) the ED is not curable, we know that, but the problems associated with it, you can deal with it. Just do it one at a time and just …. and I hope that is going to be [Name of Daughter]’s attitude

Several turns omitted:

**Table:** 

Mother:	And the only other adverse thing that anybody ever said to me was some weeks after [Daughter] was born, one of my friends said to me “I think you were very brave to have another baby, I wouldn't have done it in your situation” which I thought was horrible really. I didn't like that. Um because I had consciously decided that we could cope with another child like this if we had to.

(Interview 9: Parents with affected son, 15 years)

The mother affirms the value of life with ED (in front of her affected son) and hopes that her daughter will adopt the same perspective. She then expresses hurt and distress at the remark of a friend, who implicitly criticises her decision to have another (potentially affected) child.

In the next extract, the reproductive plans of a couple's daughter are discussed (in her absence) and the likely (stigmatising) reaction of ‘other people’ (unspecified) is reported as a powerful factor weighing against her being willing to have an affected child:

**Table:** 

Father:	You know I've been worried for [Name of Daughter]. See, she's on about later on she'll want to start a family. And I said to her, perhaps they will be able to tell when the baby's in the womb. You know, when it's small. Whether it's going to be ED and then they could terminate the pregnancy if it was.
Int:	That's certainly technically possible, yeah
Father:	I'll be able to tell her. It'll be a lot off her mind now. Telling her that.
Int:	If she wants that .. to go down that sort of road .. it would probably need organising in advance. She couldn't just turn up in her pregnancy and say she wanted it tested. It would have to be organised before.
Father:	Yeah
Int:	But I mean that sort of decision is really very personal though isn't it
Father:	Yeah
Int:	I could imagine not, some people feeing okay about that and other people not
Father:	She might go half way through it
Int:	Yeah
Father:	I mean if she did have it terminated if it was ED she might regret it. She might … you don't know how it's gonna affect a woman really do you? But she is worried about it. I know she is.

Two turns omitted.

**Table:** 

Mother:	Yeah. She said I don't think I could cope. Not with the medical side, I think it would be alright. It would be other people.
Int:	Yeah
Mother:	She'd lose her temper too quickly.
Int:	She would?
Father:	Fiery like her mother.
Mother:	Very

Interview 19. Parents discuss their daughter's reproductive plans, in the presence of their affected son aged 28 years

This discussion makes explicit the powerful influence of other people's opinions on these very personal reproductive decisions. It is not so much the consequences of the condition for a child's health but the anticipated blame of herself as the mother or stigmatisation of the child that might lead this couple's daughter to use PND to avoid having an affected child. And they express how angry such statements or behaviours from other people would make their daughter. This can be seen as an expression of solidarity with their son, present during this exchange. This confirms that it is not only professionals but also friends and acquaintances whose talk can be experienced as coercive or manipulative through its denigration of affected males. The prospect of the stigmatisation to be endured by an affected child, and the blame or courtesy stigma to which the parents might be subject, is enough to provoke concern; might it sway the reproductive decisions of family members?

### 5.4 Grandmotherly guilt

One woman expressed shock that her mother, a likely carrier of XHED, felt guilty at having transmitted the condition to her daughter and grandsons. Her account rests implicitly upon a distinction between justified moral culpability, for deliberate acts that could have been decided otherwise, and biological events in which a person unknowingly played a part while making no specific, deliberate or overtly moral decision:

**Table:** 

Mother:	I never actually think that I passed on anything, you know, I don't look at it that way. It's um, so I was quite shocked when my mother was trying to explain how guilty she felt about [Names of Two Affected Sons]. Not me, **she** felt guilty about them.

Turns omitted.

**Table:** 

Mother:	And of course when [Name of Affected Son} was born he looked so much like [Name of interviewee's deceased, probably affected, brother] that my mother went into a panic. So, she's always felt guilty about the boys … She never talked about guilt with me but I have tried to tell her if anybody should feel guilty about the boys it should be me. Because, especially [Name of Younger Affected Son] because I knowingly had him, whereas with [Name of Older Affected Son] I didn't know that I had ED. But she still has this guilt thing.

(Interview 17. Parents of two affected boys)

This mother regards herself as potentially blameworthy for having had two affected sons, with the youngest son born after she had become aware of the diagnosis in the first. However she could not accept that her own mother was blameworthy as she had been completely unaware of the situation when she had her children. This allows the listener or reader to construct the woman's underlying scheme of morality: her mother is portrayed as feeling not so much a rational moral guilt as a sense of biological responsibility for having (unknowingly) transmitted the condition both to her deceased son and through her daughter to her affected grandsons. The mother sees the awareness of choice as key to determining whether feelings of guilt would be justified, while her own mother felt burdened by the *biological responsibility* even when there had been no opportunity to have chosen a different course.

Another grandmother expressed sadness and regret at having transmitted XHED to her sons and grandsons. Whereas in the past she had simply been struggling to support her sons, the sadness and regret had been growing stronger as she witnessed her daughters reliving her own past, raising affected sons and carrier daughters and bequeathing problems to their descendants:

**Table:** 

GMother:	And erm, we just like got on with it. But getting older, as the girls got older being carriers and getting married and having their babies this is where it comes differently as being. No 'cos it affected me badly the fact that [Name of Daughter One] were having [Name of Affected Grandson] and he did have it, know what I mean. I said I was so … upset, you know what I mean? Cos I didn't want her to go through that. So, not that we don't love him any less, no, but that affected me and every time one of the girls come to have babies I was traumatised. Even [Name of Daughter Two], just had a little boy, [Name of Unaffected Grandson] and I was out me mind thinking she has a baby and he's got it ….

Several turns omitted:

**Table:** 

GMother:	But like I say it's only been the last few years when the, me own girls, you know, come to have their own children. And it's a worry really, it's worrying for them you know, not for having the babies, worrying for the girls having to cope with it, and that's, that's the only down side of it that I've had at the moment, you know.

Interview 22. Grandmother with two carrier daughters present; she had two affected sons (one deceased), two carrier daughters and one affected grandson.

These passages show the variety and complexity of attitudes to reproduction and the enmeshment of these attitudes with the participants' relationships with affected family members. The grandmother speaking here reports that her feelings have changed so that she has come to feel more concern about having transmitted the ED than she had felt until the last few years before this interview.

## 6 Discussion and conclusion

In this paper we have considered the nature of stigma in genetic disorders of visible difference and how, alongside the physical, health consequences, this may impact on feelings about reproduction in families affected by XHED. With such sex-linked disorders, the mothers of affected boys may be seen as bearing responsibility in several ways: biological responsibility for transmitting the disorder, responsibility for the practical care of affected boys and, as women are often the “kin-keepers” (Green et al., 1997), for the active communication about the condition with others in the extended family. In this study, we have found women talking about their biological responsibility as well as their moral responsibility. Even the biological responsibility can cause great sadness and be experienced as a biological guilt. The fact of disease transmission can be seen as the working out of an impersonal fate, as in a Greek tragedy in which the agent's sin may have been unwitting but is no less for that, so that retribution is demanded as well as repentance being expected.

Although the numbers are small, it appears that there may be important differences between the perspectives of women in different generations. While a mother can express regret and guilt that her son is affected by XHED, she will often also express how worthwhile his life has been (especially if he is present) and reject the choice of prenatal diagnosis and a possible termination of pregnancy to avoid having an affected son. The grandmothers of affected males may also experience the guilt and regret (Lehmann et al., 2011), perhaps even more strongly; in addition to the pain of seeing their affected sons, they may identify with their carrier daughters and regret having passed to them the burden of 'biological guilt’, comparable to the feelings of women affected by myotonic dystrophy (Faulkner and Kingston, 1998). This may be seen as an extension into biology of the social roles that shape our duties and obligations, as in the role-focused moral scheme of Dorothy Emmet (1966). The women see their biological function as carrying a deep moral responsibility that they have not lived up to. Might this be because the grandmothers initially identify strongly with their affected sons but later, as their carrier daughters become mothers, identify more fully with them and experience afresh, albeit vicariously, the burden of their daughters' reproductive decisions?

There is also a contrast between the experiences of the mothers and the sisters of affected males, with the sisters' views relayed through their mothers, because they were not usually present in the interviews. The sisters in these families have not yet made their reproductive decisions and so their judgements are not yet declared. There may be another temporal asymmetry operating: mothers of affected males report feeling hurt and offended when 'friends' express doubts about the wisdom of having had another child, which they experience as direct criticism of their most personal decisions. However, the mothers of teenage or adult males can point to their sons as fine young men living worthwhile lives but the affected sons of these men's sisters have not yet been born, and the 'success' of their future lives is therefore uncertain. It may be harder for the sisters to shrug off the pessimism because they cannot yet know how resilient their sons will be in the face of stigmatisation. We have seen that an affected man may be hurt and distressed if his (carrier) daughter elects to avoid having an affected son (Clarke, 2013): that will count as an expression of no confidence in the value of his life. An affected male could potentially be hurt by a similar decision made by his sister.

Finally, the question of guilt and responsibility may also be going through another generational shift – as with other disorders – in that the possibility of a genetic diagnosis, knowledge of genetic risk and the possibility of genetic testing have all been transformed over the last 30 years. Whereas the mothers interviewed may not have made any conscious decisions about transmitting the disorder, their daughters may find it difficult to make decisions about child-bearing without taking their genetic situation into account. Whether or not they wish to make use of genetic information in reproduction, it is now difficult to do so in ignorance: they will know that there is something to be known, even if they choose to close their eyes. Complete naïvety is no longer possible.

The delay in reporting these findings, of at least a decade for most of the interviews, is to be regretted. The findings, however, remain highly relevant. Social pressures on those affected by disabilities and impairments have not diminished over this period of social austerity in which government support for health and social care has been progressively cut back. Discrimination and stigmatisation have not evaporated.

The particular facts of inheritance of XHED frame the social experience of the family as surely as the physical effects of the gene mutation shape the physiological challenges with which the affected males have to contend. Their social experience is dominated by the stigmatisation to which they are subject and this forms the backdrop to the reproductive decisions made by their female relatives. Talk about reproduction triggers reflection about the value or worth of the lives of the affected males in the family. Discussion of this topic is very sensitive and is acknowledged as such by the women in these families; critical or indelicate talk can cause great distress or offence among the young men and also their parents. These are the 'instabilities of interaction' of which Goffman spoke. The passage of time may also affect the feelings of older women about the difficult decisions with which they can see their daughters struggling, leading them to feel a biological guilt for which they cannot be subject to any rational blame: a guilt that is biological, even tragic, but blameless.

The nature of the decisions made in these families with XHED is different from the outwardly similar decisions made in families affected by genetic disorders transmitted in a different manner. In XHED and other sex-linked disorders, those directly affected by the condition are males who do not have affected children, while those making the decisions about reproduction – deciding whether or not to have children who may be affected – are females who have spent years observing the coping strategies of their affected male relatives. There are asymmetries of gender and experience that do not apply in other contexts, which add to the burden on the women. Gender asymmetry in responsibility for reproduction is familiar from other contexts, applying not only to reproduction but also to care for children and the disabled and to the communication of genetic information within the family. Thus, it is indeed remarkable when men are subject to such feelings (Hallowell et al., 2006). In this context (XHED) the various burdens stack up on the women.

In the context of spinal muscular atrophy, the different (autosomal recessive) mode of inheritance means that the assessments of the life of one individual by another within the family works out differently, with parents evaluating the lives of affected child(ren) they have already had (Boardman, 2014a, Boardman, 2014b). Both contexts, however, resemble each other in being different from the setting of antenatal screening programmes targeted at the prevention of infants affected by Down syndrome (DS), neural tube defects (NTDs) and other disorders that are more common but much less likely to recur within a family. The societal judgements made about the worth of those with the (usually sporadic) disorders DS and NTD, and made visible in the operation of screening programmes, are replaced in the setting of single gene disorders by much more personal judgements made about the lives of those with whom family members have intimate relations over many years. The societal pressures and constraints, the possibility of stigmatisation, and the anticipated level of support for families affected by genetic disorders, will be issues for XHED families as well as families in which more familiar disorders have arisen.

We have set out the social consequences of one inherited disorder in an appropriate frame, acknowledging both the biological and social realities of life for the affected men and their female relatives. In doing this we raise the question of whether the stigma and self-blame that a carrier may anticipate she will feel, if she has an affected son, may influence her reproductive decisions. Some women, who have transmitted their carrier state to their daughters, also experience sadness and guilt for having passed to their daughters this burden of being a carrier.

Finally, we may speculate about the role of anticipated stigmatisation in reproductive decisions in other contexts. In the absence of a family history, when there is no family experience to turn to, how do couples make decisions when a structural anomaly of the foetus is found in the course of a pregnancy, such as a skeletal dysplasia causing severe short stature (Ablon, 1984)? Similarly, how do parents respond when told that an amniocentesis, carried out for other reasons, has shown that their foetus has a sex chromosome anomaly, such as Turner syndrome (45,X) or Klinefelter syndrome (47, XXY)? What will the suspicion, or demonstration, of foetal anomalies lead parents to feel towards their own foetus/child, who fails to meet the expected standard of normality?

Among the relevant considerations may be anticipation that their child will be subject to stigmatisation by others. This will be grounded in their experiences of seeing other children bullied, in the school yard or cyberspace, for minor physical features or behavioural quirks. They may themselves have been bullied for such reasons or they may even have participated in such bullying. Is it these experiences that shape the reproductive decisions of parents in relation to an unanticipated foetal abnormality in the course of a wanted pregnancy? How may one access such factors and influences, if indeed they operate in this way? How do such deep-rooted emotional responses to foetal anomalies play out in decisions about the termination of a sought-after pregnancy? This is a highly delicate area and the development of methodologies to examine these processes presents a major challenge.
